# The pre-operative physical component subscale of the 12-item Short Form Health Survey was the only factor associated with 2-year clinical outcome and satisfaction after hip abductor tendon repair

**DOI:** 10.1007/s00402-026-06449-3

**Published:** 2026-07-28

**Authors:** Jay Ebert, Peter Edwards, Sven Klinken, Greg Janes

**Affiliations:** 1 School of Human Sciences (Exercise and Sport Science), University of Western Australia, Perth, Australia; 2 HFRC Rehabilitation Clinic, Perth, Australia; 3 Curtin University, Perth, Australia; 4 Perth Radiological Clinic, Perth, Australia; 5 Perth Orthopaedic & Sports Medicine Centre, Perth, Australia

**Keywords:** Hip abductor tendon tears, Surgical repair, Clinical outcome, Satisfaction, Predictive variables

## Abstract

**Introduction:**

Surgical repair in the context of symptomatic hip abductor tears unresponsive to non-operative management has demonstrated encouraging outcomes. This study sought to investigate demographic, injury/management and post-operative factors associated with clinical outcome after hip abductor tendon repair.

**Methods:**

A total of 104 patients undergoing repair were included. Patients were assessed pre-surgery and 2-years post-operatively with the modified Harris Hip Score (mHHS), Oxford Hip Score (OHS), physical (PCS) and mental (MCS) component subscales of the 12-item Short Form Health Survey (SF-12), and satisfaction. Regression analysis assessed the contribution of pre-operative demographic (age, sex, body mass index, PCS, MCS), injury/treatment (symptom duration, prior corticosteroid injections, gluteus minimus/medius tear grade and fatty infiltration) and post-operative (hip abduction strength symmetry) variables, to the 2-year mHHS and being ‘very satisfied’.

**Results:**

Mean improvement in the mHHS and OHS from baseline to 2 years was 36.0 points (95% CI, 31.9 to 40.2; *p* < 0.001) and 17.5 points (95% CI, 15.8 to 19.3; *p* < 0.001), respectively. Seven (6.7%) patients presented with symptomatic re-tearing. Of the 97 patients with 2-year follow-up, 78 (80.4%) were ‘very satisfied’. The pre-operative SF-12 PCS was the only factor associated with the 2-year mHHS (B = 0.46; 95% CI, 0.15 to 0.77; β = 0.285; *p* = 0.004; adjusted *R*^2^ = 0.072), and being ‘very satisfied’ (OR = 1.10; 95% CI, 1.01 to 1.19; *p* = 0.021). Lower baseline SF-12 PCS scores were observed in those that presented with symptomatic re-injuries (26.7 ± 5.6 vs 34.8 ± 8.0; *p* = 0.007), though no other characteristics differed.

**Conclusions:**

All clinical outcomes improved following surgery. The pre-operative SF-12 PCS was the only variable associated with outcome and satisfaction, though this association was weak and the explanatory capacity of these models was low. This indicates that the majority of outcome variability remains unexplained by the variables assessed, further questioning the clinical significance of the pre-operative SF-12 in patient selection.

**Level of evidence:**

Level IV, prospective case series.

## Introduction

Greater trochanteric pain syndrome (GTPS) is commonly encountered in the general population [32, 46]. While gluteal tendinopathy has been recognized as a key contributor to symptomatic GTPS [20], tears of the gluteus minimus and/or medius tendons can occur. In cases of hip abductor tendon tears which are unresponsive to non-operative management, surgical repair may be undertaken with several open, endoscopic and/or augmented surgical techniques reported [3, 9, 14, 21, 23–25, 29, 31]. Following surgery, published reviews have reported improved clinical outcomes [2, 6, 10, 26–28, 33, 42, 48], with satisfaction rates ranging from 66 to 90%, [5, 36, 37, 41]. However, information is lacking on factors associated with clinical outcome and improved satisfaction after surgical repair.

Allahabadi et al. [1] recently investigated factors associated with failure at a minimum 2 years after gluteus medius/minimus repair, reporting that independent risk factors for revision surgery or not achieving either a minimal clinically important difference (MCID) or patient acceptable symptom state (PASS) included smokers, concomitant low back pain, or the presence of a limp, history of psychiatric diagnosis, and those with full-thickness tears. While not previously reported in patients undergoing hip abductor tendon repair, pre-operative health status and physical function/activity are associated with post-operative clinical outcome after other orthopaedic surgeries such as proximal hamstring tendon repair, rotator cuff repair and lower limb joint arthroplasty [11, 40, 44], supporting its inclusion in predictive modelling. Fatty gluteal muscular atrophy has been associated with worse outcomes [35], while chronic degeneration of the abductor mechanism is a major impediment to surgical success in the late repair of abductor dehiscence after total hip arthroplasty [38]. Another study reported that the degree of fatty infiltration was not associated with pain, symptoms, functional capacity, perceived improvement or satisfaction after hip abductor tendon repair [19]. Of interest, both open and endoscopic repair techniques have been reported, and a recent meta-analysis reported no statistically significant differences in pain, functional recovery and complication rates between surgical approaches [42].

A robust prospective study is currently lacking that investigates the association between patient demographics, injury variables and prior treatments, and post-operative factors, and patient outcome and satisfaction after hip abductor tendon repair. This information would provide a means of more targeted pre-operative counselling and education, specific to the patient and their individual clinical presentation, on realistic expectations after surgery. Therefore, the current study hypothesized that clinical outcomes would significantly improve from baseline to 2 years post-surgery. Secondly, it was hypothesized that certain patient demographics, injury and prior treatment variables, and post-operative factors, would be associated with 2-year clinical outcome and patient satisfaction. Finally, it was hypothesized there would be no differences in these variables between patients that had, or had not, experienced a symptomatic re-injury within the 2-year follow-up period.

## Materials and methods

**Fig. 1:**
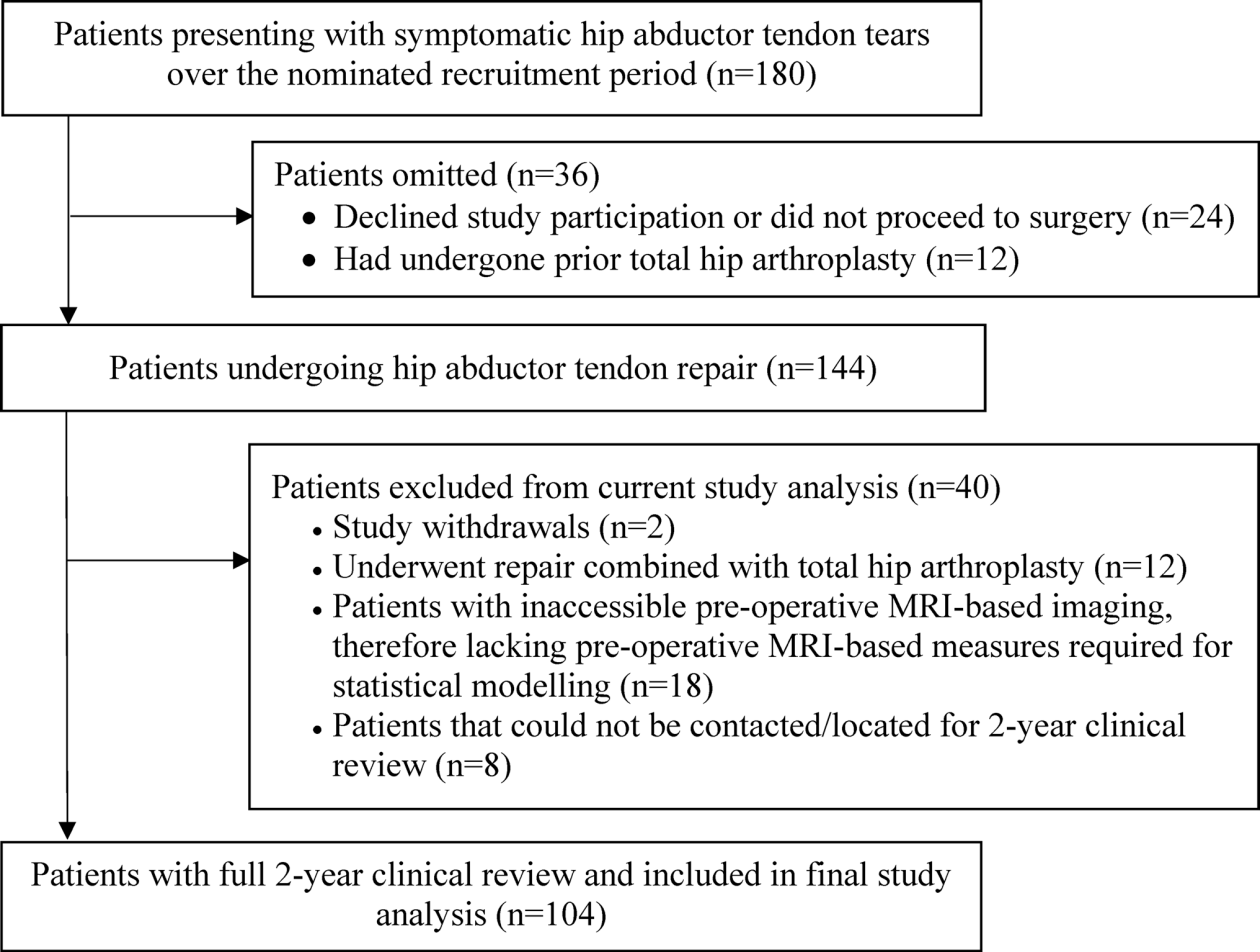
Flowchart demonstrating patients included in current study analysis

This study prospectively screened 180 patients presenting to a single surgeon between October 2012 and December 2016. Of these, 104 were included in the current analysis (Fig. 1). The indication for hip abductor tendon repair included patients presenting with symptoms of ≥ 6 months in duration and who failed non-operative treatment (generally consisting of physiotherapy and injections), with hip abductor tendon tears diagnosed on magnetic resonance imaging (MRI). Either partial or full thickness tears of gluteus minimus were evident in all cases, along with the anterior portion of gluteus medius. Ethical approval for the prospective recruitment of patients undergoing hip abductor tendon repair was provided by the Hollywood Private Hospital Human Research Ethics Committee (HPH348). The written informed consent was obtained for all patients prior to study recruitment and pre-operative data collection.

### Hip abductor tendon repair and post-operative rehabilitation

All patients underwent hip abductor tendon repair, augmented with the Ligament Augmentation and Reconstruction System (LARS, ACTOR 10, Corin Group, Cirencester, UK)) [12, 17]. Surgical procedures were undertaken under general anaesthetic and via a direct lateral approach. The trochanteric bursa was excised and the gluteus medius and minimus tendons elevated from the trochanter, with the bony footprint decorticated to expose a bleeding surface. The hip abductor tendon tear was addressed using transtendinous sutures and the broad end of a LARS ligament was sutured to the deep surface of the tendon. Two converging bone tunnels were drilled: one from the gluteus minimus foot-print on the anterior facet of the trochanter to midway through the trochanter, and the second from postero-distal on the lateral prominence of the trochanter, combining with the first tunnel. The LARS ligament was passed through the trochanter, fixed with an interference screw. The tendon was fixed to the greater trochanter with a series of interosseous sutures and a bone anchor at the superior apex of the repair. All patients participated in a graduated out-patient rehabilitation program that began at 2–3 weeks, with an initial focus on managing post-operative pain and swelling, improving mobility and increasing weight-bearing capacity. While full weight-bearing was not permitted until at least 6–8 weeks, structured exercises were initiated at 2–3 weeks post-surgery with functional weight-bearing exercises generally prescribed from 4 to 6 weeks and resisted hip abduction exercises from 8 weeks [12, 14, 16].

### Clinical outcome measures

Firstly, patient-reported outcome measures (PROMs) including the modified Harris Hip Score (mHHS) [22] and Oxford Hip Score (OHS) [8, 39] were assessed pre-operatively and at 2-years post-surgery. The minimal clinically important difference (MCID) for the mHHS after open hip abductor tendon repair has been reported as 6.2 [43] and 9.9 points [47]. While the MCID for the OHS after hip abductor tendon repair has not been reported, after total hip arthroplasty it has been reported as 5 points [39]. Specifically at 2-years, a patient satisfaction questionnaire was employed to assess the level of satisfaction with the surgery overall. A Likert response scale was employed with descriptors ‘Very Satisfied’, ‘Somewhat Satisfied’, ‘Somewhat Dissatisfied’ and ‘Very Dissatisfied’.

### Predictor variables

The variables collected and included in the current study were formulated based on our institution’s existing clinical pathway at the time of study onset, internal discussion between the orthopaedic, rehabilitation and research teams, and the very limited published evidence at the time of study onset in 2012. Patient demographics collected from all patients and included in the current analysis included age, sex and body mass index (BMI). Furthermore, duration of symptoms (DOS) was collected, as was the number of prior corticosteroid injections. Pre-operative general health was included, assessed via the 12-item Short Form Health Survey (SF-12) and including both mental (MCS) and physical (PCS) component subscales. Furthermore, peak isometric hip abduction strength was collected pre- and post-surgery, assessed using a T5 Cable Tensiometer (Pacific Scientific Company, Los Angeles, USA) [14, 18], with limb symmetry indices (LSIs) calculated. Finally, the severity of pain on a visual analogue scale (VAS) of 0–10 was reported by patients upon completion of a 30-s single leg stance (SLS) test [30].

Pre-operative MRI-based factors were also assessed. Firstly, gluteus minimus, as well as the anterior and posterior components of gluteus medius, were scored based on a 3-grade tear classification system published by Browning et al. [4], including: Grade 0 (no tear), Grade 1 (partial-thickness tears), Grade 2 (full thickness tears with < 2 cm retraction), and Grade 3 (full thickness with ≥ 2 cm retraction). Furthermore, the degree of fatty infiltration in the anterior, middle and posterior thirds of both gluteus minimus and gluteus medius was assessed as per previous published work [19]: Grade 0 (no intramuscular fat was present, Grade 1 (some fat streaks were present), Grade 2 (fat was evident, but there was less fat than muscle tissue), Grade 3 (there were equal amounts of fat and muscle tissue), and Grade 4 (there was more fat than muscle tissue). For the current analysis and, as reported previously [19], the three gradings for each part of gluteus medius and minimus (anterior, middle and posterior thirds) were summed and divided by 3 to create to mean score, ranging from 0 to 4 for each muscle. The overall gluteus medius and minimus scores were further summed and divided by 2 to create an overall combined FI score, ranging from 0 to 4. MRI-based outcomes were scored by an experienced musculoskeletal radiologist.

### Statistical analysis

Descriptive statistics were performed for baseline patient demographics, injury and treatment history, pre-operative MRI tissue characteristics, and 24-month clinical outcomes. Continuous variables were presented as mean ± standard deviation, while categorical variables were reported as frequencies and percentages. Paired *t*-tests were used to evaluate the change in clinical outcomes from baseline to 24 months post-surgery. Univariable and multivariable linear regressions were undertaken for the 24-month mHHS, while binary logistic regression analyses were employed for 24-month overall satisfaction with the surgery (‘very satisfied’ versus all other satisfaction responses). Given the skewed distribution of satisfaction responses, with no patient reporting being very dissatisfied and only two somewhat dissatisfied, overall satisfaction was dichotomized as "very satisfied" versus all other responses. As a sensitivity analysis, a proportional-odds ordinal logistic regression was undertaken across three ordered categories: very satisfied, somewhat satisfied, and dissatisfied (the somewhat dissatisfied and very dissatisfied responses combined into a single category.

For each regression model, potential pre-operative predictors were first evaluated univariably, and those displaying associations with outcomes at *p* ≤ 0.20 were included in a multivariable linear regression model. In the final step, non-significant variables (*p* > 0.05) were removed from the initial multivariable model one at a time. In the linear regression model for the 24-month mHHS, Beta coefficients, along with their 95% confidence intervals, were calculated to quantify the influence of each predictor on the dependent variable, with standardized Beta values (*β*) included in the final multivariable model. For the binary logistic regression analysis, each predictor variable was expressed as odds ratios (OR) and 95% confidence intervals. Baseline characteristics of patients who went on to present with symptomatic re-tearing were compared with those who did not using independent samples t-tests. All analyses were carried out using R (Version 4.5.1) with the lm.beta package (Version 1.7-3).

## Results

Of the full cohort (*n* = 180) that presented and were screened over the recruitment period, 104 were included in the current analysis (Fig. 1). Those that were excluded from the current analysis included those that declined study participation or did not proceed to surgery (*n* = 24), those that had undergone prior total hip arthroplasty (*n* = 12) or subsequently underwent hip abductor tendon repair with concomitant total hip arthroplasty (*n* = 12). Post-operatively, excluded patients were those that withdrew from the study prior to full 2-year clinical follow-up (*n* = 2), those that could not be contacted or located for 2-year clinical review (*n* = 8), and a series of patients for whom pre-operative MRIs could no longer be accessed (*n* = 18), therefore lacking pre-operative MRI-based measures required for statistical modelling. When comparing the baseline demographics, injury history and pre-operative clinical scores between the included cohort (*n* = 104) and the patients excluded due to inaccessible pre-operative MRIs (*n* = 18), no differences (*p* > 0.05) were identified between groups in any of the variables outlined in Table 1. Of the 104 patients who underwent hip abductor tendon repair and were prospectively enrolled, seven (6.7%) sustained a post-operative re-injury during the follow-up period. These patients had no 24-month outcome data collected and were therefore not included in the regression analyses. Of those remaining, 97 had complete clinical follow-up to 2 years post-surgery.

**Table 1:** Patient demographics, along with injury and surgery characteristics, and pre-operative magnetic resonance imaging (MRI) variables, of the patient cohort undergoing hip abductor tendon repair and included in the current analysis

	Baseline	2 years
*Patient characteristics*		
Age, years	64.6 ± 8.9	
Sex—female	98 (94.2%)	
BMI, kg/m^2^	27.9 ± 4.8	
Duration of symptoms, years	3.7 ± 3.2	
Prior cortisone injections,* n*	3.2 ± 2.0	
Operated hip—right side	53 (51.0%)	
*MRI tissue characteristics*		
Gmin tear grade (0–3)	1.4 ± 0.8	
Gmed anterior tear grade (0–3)	1.2 ± 0.8	
Gmed posterior tear grade (0–3)	0.8 ± 0.7	
FA GMed (0–4)	0.9 ± 0.8	
FA GMin (0–4)	1.4 ± 0.8	
FA combined (0–4)	1.2 ± 0.6	
*Clinical outcome*		
mHHS	55.7 ± 19.0	92.5 ± 12.9
OHS	26.3 ± 7.9	44.2 ± 4.5
SF-12 PCS	34.3 ± 8.1	46.6 ± 8.9
SF-12 MCS	48.2 ± 11.6	55.9 ± 8.6
SLS 30-s pain (VAS pain, 0–10)	4.5 ± 3.0	0.6 ± 1.2
Peak hip abductor strength LSI, %	89.9 ± 43.9	103.5 ± 15.1

Shown are means (SD) with range, or * n* (%)

*BMI* body mass index, *MRI* magnetic resonance imaging, *FA* fatty atrophy, *Gmin* gluteus minimus, *Gmed* gluteus medius, *mHHS* modified Harris Hip Score, *OHS* Oxford Hip Score, *SF-12* 12-item Short Form Health Survey, *PCS* Physical Component Subscale, *MCS* Mental Component Subscale, *SLS* single-leg stance, *VAS* visual analogue scale, *LSI* limb symmetry index

Patient characteristics, injury and treatment variables, pre-operative MRI-based measures and baseline (pre-surgery) and 2-year post-operative clinical scores of the included cohort are shown in Table 1. Mean improvement in the mHHS and OHS from baseline to 24 months was 36.0 points (95% CI, 31.9 to 40.2; *p* < 0.001) and 17.5 points (95% CI, 15.8 to 19.3; *p* < 0.001), respectively. The SF-12 PCS improved by 11.8 points (95% CI, 9.9 to 13.7; *p* < 0.001), while the MCS improved by 7.7 points (95% CI, 5.4 to 10.0; *p* < 0.001). The peak hip abductor strength LSI improved by 12.4% (95% CI, 3.2 to 21.6; *p* = 0.009). Pain reported on the VAS at completion of the 30-s SLS test decreased by 3.6 points (95% CI, 3.1 to 4.2; *p* < 0.001). Of the 97 patients with 24-month follow-up, 78 (80.4%) were ‘very satisfied’ with the surgical outcome, 17 (17.5%) were ‘somewhat satisfied’, and 2 (2.1%) were ‘somewhat dissatisfied’.

Univariable and multivariable linear regression models for the 24-month mHHS are shown in Table 2. The baseline SF-12 PCS (*p* = 0.004), baseline mHHS (p = 0.050), baseline hip abductor strength LSI (*p* = 0.059), and age (*p* = 0.146) met the univariable screening threshold of *p* ≤ 0.20. Following backward elimination, the baseline SF-12 PCS was the only independent predictor retained in the final model (B = 0.46; 95% CI, 0.15 to 0.77; *β* = 0.285; *p* = 0.004; adjusted *R*^2^ = 0.072).

**Table 2:** Univariable and multivariable linear regression models for the 2-year modified Harris Hip Score (mHHS)

Predictor variable	Univariable		Multivariable (Adjusted *R*^2^ = 0.104)		Final Multivariable Model (Adjusted *R*^2^ = 0.072)		
	B (95% CI)	*P* value	B (95% CI)	*P* value	B (95% CI)	Standardized β	*P* value
Age, years	− 0.21 (− 0.50 to 0.08)	0.146	− 0.23 (− 0.51 to 0.05)	0.112			
Sex, male	4.70 (− 6.08 to 15.48)	0.389					
BMI, kg/m^2^	− 0.23 (− 0.77 to 0.30)	0.392					
Duration of symptoms, y	− 0.30 (− 1.11 to 0.51)	0.460					
Prior cortisone injections, n	− 0.66 (− 1.99 to 0.68)	0.331					
Gmin tear grade	1.09 (− 2.06 to 4.25)	0.493					
Gmed anterior tear grade	− 1.43 (− 5.05 to 2.19)	0.435					
Gmed posterior tear grade	− 0.66 (− 4.51 to 3.19)	0.733					
FA GMed	− 1.59 (− 5.02 to 1.83)	0.357					
FA GMin	0.56 (− 2.81 to 3.93)	0.742					
FA combined	− 0.69 (− 4.69 to 3.32)	0.734					
Baseline mHHS	0.13 (− 0.00 to 0.27)	0.050					
Baseline SF-12 PCS	0.46 (0.15 to 0.77)	0.004	0.31 (-0.03 to 0.66)	0.074	0.46 (0.15 to 0.77)	0.285	0.004
Baseline SF-12 MCS	0.03 (− 0.19 to 0.25)	0.789					
Baseline 30 s SLS (0–10 pain)	− 1.01 (− 1.85 to− 0.16)	0.020	− 0.59 (− 1.51 to 0.34)	0.211			
Baseline hip abductor strength LSI, %*	0.55 (− 0.02 to 1.12)	0.059	0.45 (− 0.10 to 1.01)	0.109			

*BMI* body mass index, *Gmin* gluteus minimus, *Gmed* gluteus medius, *FA* fatty atrophy, *mHHS* modified Harris Hip Score, *SF-12* 12-item Short Form Health Survey, *PCS* Physical Component Subscale, *MCS* Mental Component Subscale, *SLS* single-leg stance, *LSI* limb symmetry index

*Strength LSI coefficients represent the change per 10 percentage-point increase in limb symmetry index

The binary logistic regression analysis for overall satisfaction is shown in Table 3. Following univariable screening and backward elimination, the baseline SF-12 PCS was the only independent predictor of being 'very satisfied' at 24 months (OR = 1.10; 95% CI, 1.01 to 1.19; *p* = 0.021), with each one-point increase associated with a 10% increase in the odds of being very satisfied (Nagelkerke *R*^2^ = 0.101). The SF-12 PCS association was essentially unchanged in the ordinal sensitivity analysis (OR = 1.09; 95% CI, 1.01 to 1.18; *p* = 0.023).

**Table 3:** Univariable and multivariable binary logistic regression models for 2-year overall satisfaction with hip abductor tendon repair surgery

Predictor variable	Univariable			Multivariable (Nagelkerke R^2^ = 0.208)			Final Multivariable Model (Nagelkerke R^2^ = 0.101)		
	*B* (SE)	OR (95% CI)	*P* value	*B* (SE)	OR (95% CI)	*P* value	*B* (SE)	OR (95% CI)	*P* value
Age, years	0.00 (0.03)	1.00 (0.95 to 1.06)	0.894						
Sex, male	− 0.78 (0.91)	0.46 (0.08 to 2.72)	0.391						
BMI, kg/m^2^	− 0.07 (0.05)	0.93 (0.84 to 1.03)	0.179	− 0.08 (0.06)	0.92 (0.81 to 1.04)	0.174			
Duration of symptoms, y	− 0.03 (0.07)	0.97 (0.84 to 1.13)	0.727						
Prior cortisone injections, n	0.06 (0.14)	1.07 (0.81 to 1.40)	0.641						
Gmin tear grade	− 0.16 (0.30)	0.85 (0.48 to 1.52)	0.583						
Gmed anterior tear grade	− 0.51 (0.34)	0.60 (0.31 to 1.17)	0.135	− 0.27 (0.39)	0.76 (0.35 to 1.65)	0.489			
Gmed posterior tear grade	− 0.52 (0.36)	0.59 (0.29 to 1.20)	0.145	− 0.54 (0.45)	0.58 (0.24 to 1.40)	0.226			
FA GMed	− 0.13 (0.33)	0.88 (0.46 to 1.67)	0.692						
FA GMin	− 0.26 (0.33)	0.77 (0.40 to 1.47)	0.426						
FA combined	− 0.27 (0.39)	0.76 (0.36 to 1.63)	0.482						
Baseline mHHS	0.01 (0.01)	1.01 (0.99 to 1.04)	0.322						
Baseline SF-12 PCS	0.09 (0.04)	1.10 (1.01 to 1.19)	0.021	0.07 (0.04)	1.08 (0.99 to 1.17)	0.088	0.09 (0.04)	1.10 (1.01 to 1.19)	0.021
Baseline SF-12 MCS	0.00 (0.02)	1.00 (0.96 to 1.05)	0.872						
Baseline 30 s SLS (0–10 pain)	− 0.18 (0.09)	0.84 (0.70 to 1.00)	0.050	− 0.08 (0.10)	0.92 (0.75 to 1.13)	0.442			
Baseline hip abductor strength LSI, %*	0.14 (0.09)	1.15 (0.96 to 1.37)	0.127	0.11 (0.09)	1.12 (0.94 to 1.34)	0.210			

*BMI* body mass index, *Gmin* gluteus minimus, *Gmed* gluteus medius, *FA* fatty atrophy, *mHHS* modified Harris Hip Score, *SF-12* 12-item Short Form Health Survey, *PCS* Physical Component Subscale, *MCS* Mental Component Subscale, *SLS* single-leg stance, *LSI* limb symmetry index

*Strength LSI coefficients represent the change per 10 percentage-point increase in limb symmetry index

Seven patients (6.7%) sustained a post-operative re-injury during the follow-up period. Patients who sustained a re-injury had significantly lower baseline SF-12 PCS scores compared to those who did not (26.7 ± 5.6 vs 34.8 ± 8.0; *p* = 0.007) and tended to have lower baseline mHHS scores (44.4 ± 14.1 vs 56.5 ± 19.1; *p* = 0.067). No other baseline characteristics differed between those who re-injured or not over the 24-month follow-up period, though given the small number of re-injuries (*n* = 7) a formal regression analysis for this outcome was not undertaken.

## Discussion

The most important finding of the current study was that while all clinical outcome measures significantly improved following surgery, the only variable that was associated with 2-year clinical outcome and satisfaction was the pre-operative patient-reported SF-12 PCS. However, this association was weak and little variance was explained by the SF-12 PCS questioning its clinical significance in patient selection. Furthermore, patients that presented with symptomatic re-tears within the 2-year follow up period reported significantly lower pre-operative SF-12 PCS scores and tended to have lower pre-operative mHHS scores.

A significant improvement in all clinical scores was observed over the 2-year period, including the mHHS and OHS, with mean improvements greater than previously reported MCIDs [39, 43, 47]. Furthermore, significant improvements were observed for the SF-12 PCS and MCS, as well as pain reported upon completion of a 30-s SLS test and the peak hip abductor strength LSI. This was in support of the first hypothesis. Prior published reviews have reported encouraging patient-reported outcomes after hip abductor tendon repair [2, 6, 10, 26–28, 33, 48], albeit improvements in functional measures (such as strength or SLS capacity) are less reported. Furthermore, of the patients that underwent 2-year clinical review (excluding the seven patients that presented with a symptomatic re-injury over the period), 97.9% were satisfied, with 80.4% ‘very satisfied’ with their surgical outcome. Patient satisfaction rates ranging from 66 to 90% have been reported after hip abductor tendon repair [5, 7, 34, 36–38, 41].

The baseline SF-12 PCS, mHHS, hip abductor strength LSI and age were all univariably associated with the 2-year mHHS, though only the pre-operative SF-12 PCS was retained in the final multivariable model. Similarly, the baseline SF-12 PCS was the only independent predictor of patients reporting being 'very satisfied' at 2 years. The reasons for the association between these 2-year outcomes and the pre-operative SF-12 PCS remain unclear, though it may be that those that perceive their physical health to be better prior to surgery may have greater self-efficacy, subsequently creating a greater commitment to the rehabilitation and recovery pathway. While this rationale remains speculative, other studies have reported an association between pre-operative health status, physical function and/or activity with post-operative clinical outcome after other procedures such as proximal hamstring tendon repair, rotator cuff repair and lower limb joint arthroplasty [11, 40, 44]. Nonetheless, while this provided support for the second hypothesis, it should be acknowledged that the association between the SF-12 PCS and 2-year clinical outcomes was weak and the low variance explained by the SF-12 limits its clinical usefulness in selecting appropriate patients for surgical intervention.

Studies reporting on factors associated with outcome after hip abductor tendon repair have generally reported on MRI-based tissue variables, such as tear grade and the degree of fatty infiltration [21, 35, 38], which the current study did evaluate. However, neither the duration of symptoms prior to surgery, the number of prior corticosteroid injections, or the tear severity or degree of fatty infill, were associated with 2-year outcome or satisfaction. Kenanidis et al. [28] proposed a treatment algorithm for the surgical treatment of hip abductor tendon tears, that was based on factors including the extent of fatty muscular infiltration and hip abductor function. Those with a Goutallier Fuchs < 2 are considered candidates for direct surgical repair, with those ≥ 2 better suited to reconstruction via a gluteus maximus flap or vastus lateralis transfer procedure. Maslaris et al. [35] investigated outcomes after arthroscopic and open repair, as well as those that had undergone prior hip arthroplasty. Worse fatty muscular degeneration scores were associated with poor outcomes with respect to the presence of a limp, patient-reported outcomes and complication rates. Fink et al. [21] also reported that in a cohort undergoing repair via osseous fixation with securement of the suture anchor by a non-resorbable collagen patch, post-operative functional results were dependent on the level of pre-operative fatty muscular degeneration. Finally, Miozzari et al. [38] reported that chronic degeneration in the abductor mechanism was a major impediment to surgical success in the late repair of abductor dehiscence after hip arthroplasty. Of interest, another study reported that in patients undergoing augmented hip abductor tendon repair, the degree of pre-operative fatty infiltration was not associated with pain, symptoms, functional capacity, perceived improvement or satisfaction at 2 years after surgery [19]. The reasons for the lack of association in the current study are unknown, though may be related to the augmented nature of the surgical procedure or influenced by the dedicated rehabilitation program all patients embarked on following surgery. In addition, the relatively low sample size included may render the current study underpowered in its ability to detect associations between pre-operative MRI-based parameters and post-operative clinical outcome. Furthermore, it is acknowledged that muscle volumetric analysis and more sophisticated imaging modalities to evaluate tendon tissue microstructure were not employed, and these may be more sensitive in detecting differences across patients in the pre- and post-operative assessment of tissue quality.

The current study reported a 6.7% re-injury rate over the 2-year period, albeit this was only reflected by patients presenting with recurrence of symptoms that subsequently returned for further review and MRI, for which the re-tear was evident. This would not account for any asymptomatic re-tears that may have been present, given that serial post-operative MRI was not standard practice. Nonetheless, while a formal regression analysis could not be undertaken given the small patient cohort that presented with symptomatic re-injury, it was interesting to note that again a lower pre-operative SF-12 PCS was evident in those patients, as was a tendency toward a lower pre-operative mHHS. However, there were no other differences in the array of pre-operative variables that differed between those that did, or did not, present with symptomatic re-injury. Allahabadi et al. [1] recently investigated factors associated with failure at a minimum 2 years after gluteus medius/minimus repair, reporting that independent risk factors for revision surgery or not achieving either a minimal clinically important difference (MCID) or patient acceptable symptom state (PASS) included smokers, those with concomitant pre-operative lower back pain, or the presence of a limp or Trendelenburg gait, history of psychiatric diagnosis, and those with full-thickness tears (particularly those with ≥ 2 cm of retraction). The current study did not evaluate these variables.

While the current study included a prospectively recruited cohort of 104 patients undergoing hip abductor tendon repair, with a comprehensive series of demographic, injury/management and post-operative factors collected, along with clinical outcomes, some limitations should be acknowledged. First, the array of endoscopic, open and/or augmented surgical procedures is appreciated, and it may be that the outcomes of the current study utilizing a particular augmented repair cannot be generalized across all repair techniques. Second, despite the array of variables included, the limited explanatory power of the multivariable model would suggest that a large proportion of outcome variability in both the mHHS and overall satisfaction is unexplained, potentially influenced by a range of other variables not assessed in the current study. This may include other health and musculoskeletal comorbidities, post-operative factors and actual diligence with rehabilitation, and patient expectations. While again it should be acknowledged that the study collected and included an array of demographic, injury-related and post-operative variables, these were based on our institution’s existing clinical pathway at that time, internal discussion between the orthopaedic, rehabilitation and research teams, and the very limited published evidence at the time of study onset in 2012. Further to this, the current study sought to investigate factors associated with 2-year clinical outcome, and it should be acknowledged that associated variables may differ when evaluating mid- and long-term clinical outcome, satisfaction and functional recovery, as well as hip abductor muscle quality, tendon integrity and/or re-injury risk.

Third, the current study employed the mHHS as the 2-year measure of clinical outcome and, while an earlier review reported that the mHHS (and HHS) and OHS were common PROMs employed in studies evaluating the outcome of hip abductor tendon repair [13], other PROMs are utilized such as the Victorian Institute for Sport Assessment for Gluteal Tendinopathy (VISA-G) score which appears responsive and more resistant to ceiling effects (compared to the mHHS and OHS) in patients undergoing hip abductor tendon repair [15]. Fourth, it is acknowledged that the exclusion of 28 patients due to post-operative study withdrawal (*n* = 2), inability to contact/locate for 2-year clinical review (*n* = 8) and the inability to access pre-operative MRI scans for scoring (*n* = 18), may introduce attrition bias. However, the inaccessibility of pre-operative MRI scans occurred at random and largely due to the initiation of study onset in 2012, also making imputation techniques difficult in these cases given the complete lack of MRI-based data for study inclusion. While a statistical comparison identified no differences in baseline demographics, injury history and pre-operative clinical scores between the included cohort and the 18 patients excluded from the analysis due to inaccessible pre-operative imaging, we are unable to clarify whether pre-operative imaging variables were the same between groups. Therefore, this may introduce an unfortunate selection bias and limit generalizability. We also acknowledge that the further exclusion of patients that re-injured may also attenuate associations between predictors and outcomes, especially if worse baseline status is linked to both re-tear and/or poorer outcomes (as outlined the cohort that re-injured did present with a lower baseline SF-12 PCS, although they presented with no pre-operative MRI-based differences). Unfortunately, 2-year clinical scores could not be collected in this cohort, hence their exclusion.

Finally, the study employed a statistical approach of variable selection in the final multivariable model based on findings of a univariable analysis. It is acknowledged that other options exist [45]. However, the approach employed is consistent with pre-existing studies in these clinical populations, and we feel the risk of the stepwise regression methodology employed increasing the possibility of model instability and overfitting was reduced in the current study given the low model complexity (only a single variable was retained in the final model) and low variance explained, with other approaches unlikely to yield a different conclusion. Nonetheless, it must be acknowledged that the use of univariable pre-screening followed by backward elimination is a potential source of bias rather than a methodological convention, and the pre-operative SF-12 PCS coefficient should therefore be interpreted with caution.

## Conclusion

Clinical outcome measures significantly improved following surgery. However, the only variable that was associated with 2-year clinical outcome and satisfaction was the pre-operative SF-12 PCS. However, this association was weak and little variance was explained by the SF-12 PCS questioning its clinical significance in patient selection. Based on these outcomes, it would appear reasonable to offer surgical intervention to patients presenting with symptomatic hip abductor tendon tears failing non-operative management, given the outcomes observed and lack of factors (including pre-operative MRI-based tissue variables) associated with 2-year outcome and satisfaction.

## Data Availability

Data has not been made publicly available, though data sets generated during the current study can be made available from the corresponding author on reasonable request.

## Abbreviations

PROMs: Patient-reported outcome measures
mHHS: Modified Harris Hip Score
OHS: Oxford Hip Score
SF-12: 12-Item Short Form Health Survey
PCS: Physical component subscale
MCS: Mental component subscale
MCID: Minimal clinically important difference
MRI: Magnetic resonance imaging
SLS: Single leg stance
BMI: Body mass index
LSI: Limb symmetry index
